# Reticular pseudodrusen load is associated with an increased risk of stroke

**DOI:** 10.1136/bmjophth-2025-002481

**Published:** 2026-07-20

**Authors:** Roy Schwartz, Abraham Olvera-Barrios, Alasdair N. Warwick, Hagar Khalid, Sumita Phatak, Mahima Jhingan, Coen de Vente, Philippe Valmaggia, Sandra Liakopoulos, Yanling Ouyang, Clara I. Sanchez-Guiterrez, Catherine Egan, Adnan Tufail

**Affiliations:** 1 NIHR Biomedical Research Centre, Moorfields Eye Hospital NHS Foundation Trust & UCL Institute of Ophthalmology, London, UK; 2 Moorfields Eye Hospital NHS Foundation Trust, London, UK; 3 Apellis, a subsidiary of Biogen, London, UK; 4 University College London, Institute of Cardiovascular Science, London, UK; 5 Quantitative Healthcare Analysis (qurAI) Group, Informatics Institute, University of Amsterdam, Amsterdam, Netherlands; 6 Amsterdam UMC location University of Amsterdam, Biomedical Engineering and Physics, Amsterdam, Netherlands; 7 Department of Biomedical Engineering, University of Basel, Basel, Switzerland; 8 Department of Ophthalmology, University Hospital Basel, Basel, Switzerland; 9 Cologne Image Reading Center, Department of Ophthalmology, Faculty of Medicine and University Hospital Cologne, University of Cologne, Cologne, Germany; 10 Department of Ophthalmology, Goethe University, Frankfurt, Germany; 11 Southampton Eye Unit, University Hospital Southampton, Southampton, UK

**Keywords:** Epidemiology, Imaging, Macular Degeneration, Retina

## Abstract

**Background/aims:**

Reticular pseudodrusen (RPD) are increasingly recognised as a distinct phenotype in the age-related macular degeneration (AMD) disease spectrum. This study investigates the association between RPD and cardiovascular outcomes, specifically myocardial infarction (MI) and stroke in the UK Biobank (UKBB).

**Methods:**

Retrospective analysis of UKBB participants (n=2010). A validated deep learning framework identified and quantified subjects with RPD, drusen and controls on optical coherence tomography. Five retina specialists validated the artificial intelligence findings. Multivariable logistic regression models assessed the association between RPD/drusen and stroke, MI and combined MI/stroke, adjusting for age, sex, high-density lipoprotein cholesterol/low-density lipoprotein cholesterol ratio and smoking history.

**Results:**

The study cohort included 71 subjects with pure RPD, 401 with pure drusen, 368 with both and 1170 controls. Univariable analysis showed that for every 20 RPD lesions, the OR for stroke increased by 1.02 (95% CI 1.00 to 1.04, p=0.028). The mean number of RPD per patient in this cohort was 199, indicating an increased stroke risk of 20%. Multivariable analysis showed a significant association between RPD load and stroke risk (OR 1.02, 95% CI 1.00 to 1.04, p=0.042) after adjustment for confounders. No significant association was found between drusen and stroke or between RPD/drusen and MI.

**Conclusion:**

Increased RPD load is associated with a higher risk of stroke, independent of traditional cardiovascular risk factors. Drusen did not have similar associations, suggesting that RPD may represent a distinct disease entity. Evaluating cardiovascular risk in patients with numerous RPD might be advisable. Further prospective studies are needed to validate these findings and explore the underlying mechanisms.

**WHAT IS ALREADY KNOWN ON THIS TOPIC**

- Small-scale studies have suggested associations between reticular pseudodrusen (RPD) and cardiovascular disease, but large epidemiological studies examining drusen-associated age-related macular degeneration (AMD) have failed to demonstrate consistent cardiovascular associations. RPD are increasingly recognised as anatomically, biochemically and genetically distinct from drusen, suggesting they may represent a separate disease entity with unique systemic associations.

**WHAT THIS STUDY ADDS**

- This large UK Biobank study demonstrates that increased RPD load is independently associated with stroke risk, with every 20 RPD lesions conferring a 2% increased odds of stroke. No similar association was found between drusen and stroke or between either lesion type and myocardial infarction, supporting the hypothesis that RPD represents a distinct pathological entity separate from drusen-associated AMD.

**HOW THIS STUDY MIGHT AFFECT RESEARCH, PRACTICE OR POLICY**

- These findings suggest that patients with high RPD burden may benefit from cardiovascular risk assessment and stroke prevention strategies. The quantification of RPD load using artificial intelligence-based tools could potentially be incorporated into clinical risk stratification algorithms, though prospective validation studies are needed to establish causality and clinical utility before implementation in routine practice.

## Introduction

 Age-related macular degeneration (AMD) affects the macular region of the retina, leading to central vision loss in older individuals. It ranks as the third leading cause of severe, irreversible vision loss globally.^1^ AMD is conventionally characterised by the presence of drusen—acellular, lipid-rich deposits beneath the retinal pigment epithelium (RPE).^2^ The presence and size of drusen are key factors in the current AMD classification system.^3^

Thanks to advancements in retinal imaging, reticular pseudodrusen (RPD, also known as subretinal drusenoid deposits) are now recognised as an additional phenotype in patients with AMD.^4^

A growing body of evidence highlights the differences between drusen and RPD. Anatomically, drusen are located below the retinal pigment epithelium (RPE), while RPD are found above it.^5^ Numerous studies have demonstrated that the presence of RPD in AMD eyes is a risk factor for rapid progression to advanced stages of AMD, including geographic atrophy (GA) and type 3 macular neovascularisation (MNV).^6 7^ Moreover, these phenotypes differ in their biochemical compositions. Drusen contain both esterified and non-esterified cholesterol, while RPD predominantly contain unesterified cholesterol.^8^ A recent publication also revealed that RPD are rich in lysolipids, a characteristic not found in drusen.^9^ Small-scale studies have suggested links between RPD and coronary artery disease (CAD), lower levels of HDL and stroke.^10^ Subsequent work has further refined this association, identifying specific high-risk vascular diseases (HRVDs) that may compromise ocular perfusion, including myocardial dysfunction, valvular heart disease and internal carotid artery stenosis, as particularly associated with RPD, with the hypothesis that RPD formation results from ocular hypoperfusion consequent to these conditions.^11^ Supporting this, a prospective study demonstrated that choroidal thinning precedes incident RPD.^12^

However, no consistent associations have been observed with drusen-associated AMD in larger studies.^13^,^15^ Additionally, meta-analyses have not established a connection between AMD and cardiovascular diseases (CVD) or stroke.^16 17^ Genome-wide association studies have further examined the relationship between various AMD-related genes and RPD. They revealed an association between the age-related maculopathy susceptibility 2 (ARMS2) risk allele and individuals with RPD, but not with the complement factor H (CFH) risk allele. Both alleles are known to be linked to drusen-associated AMD.^18 19^ RPD can also occur in conditions where drusen are not described, such as pseudoxanthoma elasticum^20^ and vitamin A deficiency.^21^

Given these differences, it is possible that RPD represents a distinct disease entity, separate from drusen, via a unique biological pathway, with its own genetic and potentially systemic associations. To explore this hypothesis, we examined associations of adverse cardiovascular outcomes, specifically focusing on myocardial infarction (MI) and stroke, between RPD and drusen among subjects from the UK Biobank (UKBB) study.

## Materials and methods

### The UKBB

The UKBB study is a large, multisite, community-based cohort study aimed at enhancing the prevention, detection and treatment of a broad spectrum of serious and life-threatening diseases. It encompasses data from 500 000 volunteer participants, aged 40–69, who were recruited between 2006 and 2010 throughout the UK. All UK residents in this age group, registered with the National Health Service and living within 25 miles of one of the 22 assessment centres, were invited to participate. The UKBB project ID associated with this research is 60 078. Participants who withdrew their consent were excluded from the study.

In the UKBB, 67 687 participants underwent optical coherence tomography (OCT) and colour fundus photography (CFP) imaging at six centres (Sheffield, Liverpool, Hounslow, Croydon, Birmingham and Swansea). Images were captured using the Topcon 3D OCT 1000 Mark II (Topcon, Japan) under mesopic conditions without pupil dilation using the three-dimensional macular volume scan (512 horizontal A-scans/B-scan; 128 B-scans in a 6×6 mm raster pattern).

### Study population

A deep learning framework was used to identify subjects with RPD, drusen and control groups in the UKBB dataset. Details of the framework have been published previously.^22^ Briefly, an image classifier identifies ungradable OCT volumes. This is followed by a deep ensemble model that detects out-of-distribution data, further identifying ungradable volumes. After removing these, an additional classifier evaluates the gradable OCT volumes to identify subjects with drusen, RPD and control groups. The process includes a fourth step, which uses a semantic segmentation model to quantify the number of RPD and drusen instances within each B-scan.

The artificial intelligence (AI) framework was deployed across the entire UKBB OCT dataset to identify subjects with drusen, those with RPD and a control group without these phenotypes. Subjects identified by the AI framework were included in the study based on the following criteria. (1) Age of 60 years or older. (2) Presence of at least five instances of RPD or drusen per eye to exclude cases with minimal lesions that may be due to natural variation. (3) For those with drusen, the size had to exceed 63 μm in at least one eye, ensuring the exclusion of subjects with only drupelets, in line with the classification of at least early AMD according to the Beckman classification. To determine the correct size of drusen, we calculated the maximum diameter of drusen in any direction (Feret diameter) in the en face plane.

Two retina specialists (HK and RS) graded the cases identified by the AI framework for the presence of RPD, drusen or both. Multimodal imaging, including OCT and CFP, was used in the validation process. Cases with late AMD (GA or MNV) were excluded, as the resulting changes to the outer retina may limit the accuracy of lesion quantification. Any disagreement between graders was adjudicated by a senior grader (AT).

Four retina specialists (AT, SP, MJ and RS) validated the control group. Control status was confirmed through multimodal imaging assessments, including OCT volume and CFP, ensuring no drusen or RPD was present. CFPs complemented OCT scans to detect any drusen or RPD outside the field of view of OCT scans. Subjects with ungradable CFPs were excluded, as were those with imaging artefacts on either OCT or CFP that hindered confirming their control status, such as shadowing on CFPs or artefacts affecting the outer retina on OCT.

After validating the cases, the total number of RPD and drusen identified by the AI model across all B-scans of both eyes for each patient was summed. This total represented the lesion count per patient.

### Validation of AI lesion quantification

To validate the accuracy of the AI model’s lesion counting, two experienced retina specialists (authors SL and YO) independently annotated drusen and RPD lesions (stages 2, 3 and 4 combined) on a subset of 67 B-scans. Lesion counts were then derived from these annotations by identifying connected components using the Label function of the scikit-image Python library, which finds connected components in binary images. Intergrader agreement and agreement between each grader and the model were assessed using the intraclass correlation coefficient (ICC) for absolute agreement.

### Health outcomes

The primary outcome was stroke, which was recorded through electronic health record (EHR) linkage and participant self-reported health conditions (algorithmically defined outcomes including stroke, intracerebral haemorrhage and/or subarachnoid haemorrhage definitions). MI (defined as MI, ST elevation MI and/or non-ST elevation MI) and cardiovascular adverse events, defined as MI and/or stroke, were examined as secondary outcomes.

### Assessment of cardiovascular risk factors

Diagnoses of type 1 and type 2 diabetes, hypertension and atrial fibrillation were ascertained using UKBB algorithmically defined outcomes derived from linked health records and participant reports. Smoking history was gathered through a questionnaire, focusing on two variables: ‘Current Tobacco Smoking’ and ‘Past Tobacco Smoking’. These variables were presented as questions to the participants. Only those who reported smoking currently or in the past, on most or all days (as opposed to occasionally or never), were considered to have a positive smoking history in this analysis.

The high-density lipoprotein cholesterol (HDL-C) to low-density lipoprotein cholesterol (LDL-C) ratio (HDL-C/LDL-C) was used to adjust for the protective and atherogenic lipoprotein balance, given evidence of associations with all-cause mortality and major adverse cardiovascular events in the UKBB.^23^

### Statistical analysis

We undertook analyses using logistic regression for stroke, adjusting for age at baseline, sex, number of RPD (Model_Stroke_ 1), prevalent diabetes, prevalent hypertension, prevalent atrial fibrillation (Model_Stroke_ 2), HDL-C/LDL-C ratio (Model_Stroke_ 3) and smoking history (Model_Stroke_ 4). Multivariable models following the same modelling strategy were used to test for MI (Model_MI_ 1–4) and MI/stroke (Model_CVD_ 1–4) associations. All models are presented without RPD as online supplemental tables 1,2. Statistical analyses were undertaken using R (V.4.2.2).^24^

### Patient and public involvement

Patients and members of the public were not involved in the design, conduct, reporting or dissemination plans of this research.

## Results

**Figure 1:**
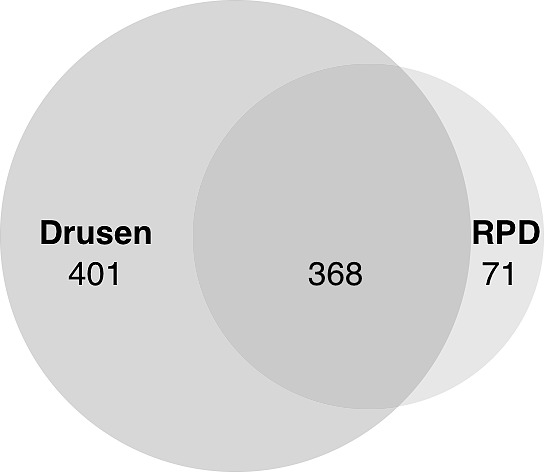
Venn diagram showing the distribution of subjects among cases. RPD, reticular pseudodrusen.

A total of 2010 subjects were validated by the graders for inclusion in the study. This group comprised 769 subjects with drusen, among whom 401 had drusen without RPD (referred to as ‘pure drusen’); 439 subjects with RPD, with 71 having only RPD without drusen (‘pure RPD’) and 1170 controls. The Venn diagram in figure 1 shows the distribution of subjects across the case groups. The mean number of RPD for participants with RPD was 199 (range 5–2834), and the mean number of drusen for participants with drusen was 394 (range 7–3717).

### Validation of AI lesion quantification

For drusen lesion counts, intergrader agreement between the two graders was good (ICC 0.79, 95% CI 0.73 to 0.83). The model achieved moderate agreement with grader 1 (ICC 0.72, 95% CI 0.60 to 0.80) and grader 2 (ICC 0.72, 95% CI 0.64 to 0.88). For RPD stages 2, 3 and 4 lesion counts, intergrader agreement was moderate (ICC 0.69, 95% CI 0.60 to 0.75), with the model achieving comparable moderate agreement with grader 1 (ICC 0.65, 95% CI 0.56 to 0.72) and grader 2 (ICC 0.69, 95% CI 0.59 to 0.77), demonstrating performance consistent with human-level accuracy.

### Demographic characteristics

Demographic characteristics of the subjects are presented in table 1. The median age for the entire group was 64 years (IQR 61–66). The pure RPD group was slightly older, with a median age of 66 years (IQR 63–68). Of the subjects, 55% (1105/2010) were female, and 96% (1925/2010) were white.

**Table 1:** Demographic characteristics

Characteristic	Overall, n=2010	Pure RPD, n=71	Pure drusen, n=401	RPD and drusen, n=368	Control, n=1170
Stroke	70 (3.5%)	3 (4.2%)	15 (3.7%)	15 (4.1%)	37 (3.2%)
Myocardial infarction	114 (5.7%)	7 (9.9%)	25 (6.2%)	15 (4.1%)	67 (5.7%)
Major adverse cardiovascular event	174 (8.7%)	10 (14%)	38 (9.5%)	27 (7.3%)	99 (8.5%)
Age at baseline	63.4 (4.2)	63.9 (6.8)	63.6 (4.7)	62.7 (5.9)	63.6 (2.9)
Sex					
Female	1105 (55%)	35 (49%)	238 (59%)	177 (48%)	655 (56%)
Male	905 (45%)	36 (51%)	163 (41%)	191 (52%)	515 (44%)
Smoking history					
Never	1300 (65%)	47 (66%)	251 (63%)	218 (59%)	784 (67%)
Previous	610 (30%)	21 (30%)	127 (32%)	123 (33%)	339 (29%)
Current	100 (5.0%)	3 (4.2%)	23 (5.7%)	27 (7.3%)	47 (4.0%)
Diagnosis of diabetes mellitus	122 (6.1%)	7 (9.9%)	25 (6.2%)	25 (6.8%)	65 (5.6%)
Duration of diabetes	7 (10)	7 (9)	8 (12)	6 (4)	8 (11)
Diagnosis of hypertension	1053 (52%)	40 (56%)	225 (56%)	196 (53%)	592 (51%)
Diagnosis of atrial fibrillation	224 (11%)	9 (13%)	46 (11%)	40 (11%)	129 (11%)
Total cholesterol (mmol/L)*	5.69 (1.19)	5.60 (1.01)	5.59 (1.20)	5.56 (1.18)	5.77 (1.20)
HDL-C (mmol/L)*	1.50 (0.39)	1.47 (0.35)	1.51 (0.37)	1.52 (0.44)	1.50 (0.39)
LDL-C (mmol/L)*	3.52 (0.89)	3.43 (0.77)	3.43 (0.89)	3.40 (0.90)	3.59 (0.90)
HDL-C/LDL-C*					
<0.4	796 (44%)	36 (52%)	149 (42%)	137 (41%)	474 (45%)
0.4 to <0.6	735 (41%)	23 (33%)	144 (40%)	130 (39%)	438 (42%)
≥0.6	281 (16%)	10 (14%)	64 (18%)	69 (21%)	138 (13%)
Ethnicity					
White	1925 (96%)	67 (94%)	386 (96%)	347 (94%)	1125 (96%)
Black	21 (1.0%)	0 (0%)	3 (0.7%)	8 (2.2%)	10 (0.9%)
Asian	25 (1.2%)	2 (2.8%)	3 (0.7%)	4 (1.1%)	16 (1.4%)
Mixed	13 (0.6%)	1 (1.4%)	3 (0.7%)	3 (0.8%)	6 (0.5%)
Chinese	2 (<0.1%)	0 (0%)	0 (0%)	0 (0%)	2 (0.2%)
Other	18 (0.9%)	1 (1.4%)	2 (0.5%)	6 (1.6%)	9 (0.8%)
Missing	6 (0.3%)	0 (0%)	4 (1.0%)	0 (0%)	2 (0.2%)
Body mass index	27.6 (4.5)	27.7 (5.1)	28.0 (4.6)	27.8 (5.3)	27.4 (4.2)

Mean (SD) for continuous variables.

Count (column %) for categorical variables.

* A total of 194/2010 (9.7%) participants with missing values for HDL-C, 123/2010 (6.1%) participants with missing values for LDL-C, 198/2010 (9.9%) participants with missing values for HDL-C/LDL-C and 11/2010 (0.5%) participants with missing values for body mass index.

HDL-C, high-density lipoprotein cholesterol; LDL-C, low-density lipoprotein cholesterol; RPD, reticular pseudodrusen.

A total of 114 (5.7%) participants had a history of MI. Notably, this percentage doubled to 9.9% in the group with pure RPD. Additionally, 70 (3.5%) participants had a history of stroke, with a similar distribution across the groups.

In terms of cardiovascular risk factors, 610 (30%) had a history of tobacco smoking, and 100 (5%) admitted to currently smoking. A total of 11% had a diagnosis of atrial fibrillation. For the total cohort, the mean direct LDL-C was 3.51 mmol/L (IQR 2.89–4.09), considered borderline high; the mean triglyceride level was 1.50 mmol/L (IQR 1.10–2.03), considered to be within normative limits; the mean HDL-C for the total cohort was 1.46 (IQR 1.22–1.73), considered normal but not high.^25^

### Associations of RPD with major cardiovascular events

The number of RPD was significantly associated with stroke (table 2). Every 20-unit increase in RPD per patient showed a 1.02 OR for stroke (95% CI 1.002 to 1.036 (p=0.028)). Multivariable models adjusting for age, sex (Model_Stroke_ 1, OR 1.02, p=0.049) and prevalent diabetes, hypertension, atrial fibrillation and HDL-C/LDL-C (Model_Stroke_ 3; OR 1.02, p=0.042) showed a stable significant association of the number of RPD with stroke. This association was not evidenced after the introduction of smoking history (Model_Stroke_ 4; OR 1.02, p=0.058). When compared with females, males showed higher odds of stroke (OR 1.99; 95% CI 1.23 to 3.28, p=0.006). No significant associations were identified for drusen or age. Lipid levels were additionally modelled as part of a sensitivity analysis, with total cholesterol and LDL-C as continuous covariates instead of the HDL-C/LDL-C ratio (online supplemental table 3). This did not materially alter the estimate for RPD and stroke (OR 1.02, 95% CI 1.00 to 1.04; p=0.047)

**Table 2:** Raw and mutually adjusted OR for stroke

Characteristic	Univariable		Model 1*		Model 2*		Model 3†		Model 4†	
	OR (95% CI)	P value	OR (95% CI)	P value	OR (95% CI)	P value	OR (95% CI)	P value	OR (95% CI)	P value
RPD (per 20)	1.02 (1.00 to 1.04)	**0.028**	1.02 (1.00 to 1.03)	**0.049**	1.02 (1.00 to 1.04)	**0.037**	1.02 (1.00 to 1.04)	**0.042**	1.02 (1.00 to 1.04)	0.058
Drusen (per 5)	1.00 (1.00 to 1.00)	0.8	–		–		–		–	
Age	1.03 (0.97 to 1.10)	0.3	1.03 (0.97 to 1.10)	0.3	1.00 (0.94 to 1.06)	>0.9	1.00 (0.94 to 1.07)	>0.9	0.99 (0.93 to 1.07)	0.9
Sex	–		–		–		–		–	
Female	1.00		1.00		1.00		1.00		1.00	
Male	1.99 (1.23 to 3.28)	**0.006**	1.94 (1.20 to 3.21)	**0.008**	1.64 (1.00 to 2.74)	0.053	1.97 (1.14 to 3.45)	**0.016**	1.87 (1.08 to 3.31)	**0.027**
Diagnosis of diabetes	–		–		–		–		–	
No	1.00		–		1.00		1.00		1.00	
Yes	1.76 (0.72 to 3.69)	0.2	–		1.07 (0.43 to 2.32)	0.9	0.96 (0.35 to 2.20)	>0.9	0.92 (0.34 to 2.12)	0.9
Diagnosis of hypertension	–		–		–		–		–	
No	1.00		–		1.00		1.00		1.00	
Yes	3.18 (1.85 to 5.77)	**<0.001**	–		2.55 (1.46 to 4.70)	**0.002**	2.44 (1.34 to 4.71)	**0.005**	2.34 (1.28 to 4.52)	**0.008**
Diagnosis of atrial fibrillation	–		–		–		–		–	
No	1.00		–		1.00		1.00		1.00	
Yes	4.86 (2.88 to 8.03)	**<0.001**	–		4.04 (2.34 to 6.83)	**<0.001**	3.96 (2.22 to 6.91)	**<0.001**	3.82 (2.13 to 6.69)	**<0.001**
HDL-C/LDL-C	–		–		–		–		–	
<0.4	1.00		–		–		1.00		1.00	
0.4 to <0.6	1.78 (0.99 to 3.28)	0.059	–		–		1.91 (1.05 to 3.59)	**0.038**	1.92 (1.05 to 3.60)	**0.037**
≥0.6	2.44 (1.20 to 4.90)	**0.013**	–		–		2.56 (1.22 to 5.32)	**0.012**	2.61 (1.24 to 5.43)	**0.010**
Smoking history	–		–		–		–		–	
Never	1.00		–		–		–		1.00	
Previous	1.94 (1.19 to 3.16)	**0.007**	–		–		–		1.62 (0.94 to 2.79)	0.080
Current	0.72 (0.12 to 2.39)	0.6	–		–		–		0.63 (0.10 to 2.19)	0.5

Model 1 adjusts for age, sex and number of drusen. Model 2 adjusts as model 1 plus a diagnosis of hypertension, diabetes, and atrial fibrillation. Model 3 adjusts as model 2 plus HDL-C/LDL-C and model 4 adjusts as model 3 plus smoking history.

OR >1.00 implies greater odds of stroke. Bold p values represent statistically significant results.

* Number of observations=2010.

† Number of observations=1812.

HDL-C, high-density lipoprotein cholesterol; LDL-C, low-density lipoprotein cholesterol; RPD, reticular pseudodrusen.

Analyses evaluating the associations with MI did not identify significant associations for either RPD or drusen (table 3).

**Table 3:** Raw and mutually adjusted OR for myocardial infarction

Characteristic	Univariable		Model 1*		Model 2*		Model 3†		Model 4†	
	OR (95% CI)	p-value	OR (95% CI)	p-value	OR (95% CI)	p-value	OR (95% CI)	p-value	OR (95% CI)	p-value
RPD (per 20)	0.99 (0.95 to 1.01)	0.5	0.99 (0.95 to 1.01)	0.4	0.98 (0.95 to 1.01)	0.3	0.99 (0.95 to 1.01)	0.4	0.98 (0.95 to 1.01)	0.3
Drusen (per 5)	1.00 (1.00 to 1.00)	0.7	–		–		–		–	
Age	1.07 (1.02 to 1.13)	**0.010**	1.07 (1.01 to 1.13)	**0.016**	1.04 (0.99 to 1.10)	0.13	1.05 (0.99 to 1.12)	0.091	1.05 (0.99 to 1.12)	0.11
Sex	–		–		–		–		–	
Female	1.00		1.00		1.00		1.00		1.00	
Male	3.05 (2.04 to 4.66)	**<0.001**	3.04 (2.03 to 4.64)	**<0.001**	2.55 (1.69 to 3.93)	**<0.001**	2.84 (1.81 to 4.56)	**<0.001**	2.55 (1.61 to 4.11)	**<0.001**
Diagnosis of diabetes	–		–		–		–		–	
No	1.00		–		1.00		1.00		1.00	
Yes	4.01 (2.35, 6.60)	**<0.001**	–		2.47 (1.41, 4.17)	**0.001**	2.06 (1.10, 3.66)	**0.018**	1.91 (1.02, 3.42)	**0.034**
Diagnosis of hypertension	–		–		–		–		–	
No	1.00		–		1.00		1.00		1.00	
Yes	4.07 (2.58 to 6.69)	**<0.001**	–		3.10 (1.94 to 5.15)	**<0.001**	2.86 (1.74 to 4.90)	**<0.001**	2.72 (1.65 to 4.67)	**<0.001**
Diagnosis of atrial fibrillation	–		–		–		–		–	
No	1.00		–		1.00		1.00		1.00	
Yes	2.98 (1.88 to 4.60)	**<0.001**	–		2.10 (1.30 to 3.33)	**0.002**	2.07 (1.23 to 3.38)	**0.004**	1.98 (1.17 to 3.24)	**0.008**
HDL-C/LDL-C	–		–		–		–		–	
< 0.4	1.00		–		–		1.00		1.00	
0.4 to <0.6	1.72 (1.09 to 2.76)	**0.021**	–		–		1.71 (1.06 to 2.79)	**0.028**	1.73 (1.07 to 2.83)	**0.026**
≥0.6	1.99 (1.11 to 3.51)	**0.018**	–		–		2.01 (1.09 to 3.66)	**0.023**	2.02 (1.09 to 3.68)	**0.024**
Smoking history	–		–		–		–		–	
Never	1.00		–		–		–		1.00	
Previous	2.76 (1.84 to 4.14)	**<0.001**	–		–		–		1.98 (1.27 to 3.10)	**0.003**
Current	3.72 (1.82 to 7.06)	**<0.001**	–		–		–		2.56 (1.14 to 5.33)	**0.016**

Model 1 adjusts for age, sex and number of drusen. Model 2 adjusts as model 1 plus a diagnosis of hypertension, diabetes, and atrial fibrillation. Model 3 adjusts as model 2 plus HDL-C/LDL-C and model 4 adjusts as model 3 plus smoking history.

OR >1.00 implies greater odds of myocardial infarction. Bold p values represent statistically significant results.

* Number of observations=2010.

† Number of observations=1812.

HDL-C, high-density lipoprotein cholesterol; LDL-C, low-density lipoprotein cholesterol; RPD, reticular pseudodrusen.

## Discussion

In this study, we found that for every 20 RPD lesions, the risk of stroke increased by 2%. With a mean of 199 lesions in our study population, this results in an average 20% increased risk of stroke in our cohort. We did not find a similar association for drusen. Additionally, we identified no associations between RPD or drusen and the risk of MI.

Several studies, both clinical small-scale and large epidemiological studies, have examined the link between RPD and CVD. In a recent study, Thomson *et al* explored this association by comparing 62 subjects with RPD (29 of whom had pure RPD) to 64 subjects with only soft drusen and no RPD. In their analysis, they demonstrated that individuals with RPD had a significantly higher likelihood of CVD or stroke, a finding that remained significant after comprehensive adjustment for demographic, social, medical and genetic variables.^10^ Although our study did not identify a direct association with CVD through categorical analysis, examining the number of RPD allowed us to establish an association between stroke and RPD load. This inconsistency may have been a result of low statistical power due to the differing event rates observed in the studies. In Thomson *et al*’s research, 51 out of 126 participants (40%) experienced CVD or stroke, while the event rate in our study was lower (3.5% for stroke and 5.7% for myocardial infarction).

In a similar study by Cymerman *et al*, patients were divided into two categories: those with CAD and those without. The study revealed that RPD occurred significantly more often in patients with CAD compared with those without (RR=2.1, 95% CI 1.08 to 3.95, p=0.03). However, no significant link was found between soft drusen and CAD.^26^ Although the study did not explicitly detail the distribution of pure versus mixed RPD, the discussion implied such a distinction within the cohort. In contrast, in an earlier study by the same group examining the association of RPD with CVD, all participants had drusen, and none had pure RPD. No significant difference was found in MI occurrences between patients with and without RPD, noting only a trend towards an association with angina. Stroke risk was not assessed.^27^ Likewise, other clinic-based studies have failed to demonstrate an association between RPD and CVD in cases where RPD were present alongside drusen-associated AMD.^28^,^30^

Importantly, these prior studies examined CVD broadly rather than the specific HRVDs associated with reduced perfusion. As Ledesma-Gil *et al* and Smith *et al* have emphasised, the absence of a general CVD-RPD association does not preclude specific associations with conditions that compromise ocular blood flow.^11 31^ Our finding of an association with stroke, which can result from internal carotid artery stenosis affecting both cerebral and ocular perfusion, aligns with this more specific hypothesis.

The same applies to large epidemiological studies. The Melbourne Collaborative Cohort Study involved 87 subjects with RPD out of a total cohort of 21 130. Among these, only six (0.04%) exhibited pure RPD, indicating a predominantly AMD population. In this group, only a trend towards an association with a history of MI or stroke in the RPD group was observed.^32^ In the Rotterdam Study, RPD were identified in 137 participants (4.9%). Of these, only five (0.18%) had pure RPD. No increased association between RPD and drusen was found in relation to hypertension, and other atherosclerotic CVD risks were not analysed.^33^ Lastly, the age-related eye disease study 2 exclusively investigated RPD within the context of AMD (ie, drusen with or without RPD). The study did not find any significant differences among participants, with or without RPD, for a history of hypertension or angina.^34^

The aforementioned findings in the literature support the hypothesis that RPD may be considered a distinct disease entity, separate from drusen-associated AMD. It seems that only by separating pure RPD from drusen-associated AMD was the association uncovered, both in our work and in others’.

The differential association of RPD, but not drusen, with stroke extends the evidence for distinct pathological processes underlying these lesions. As outlined in the Introduction section, RPD and drusen differ in their anatomical location, biochemical composition and progression patterns to late AMD. We have recently demonstrated that they also have distinct genetic architectures, with RPD associated with the ARMS2/HTRA1 risk allele but not the CFH variants characteristic of drusen-associated AMD.^18^ The present finding of divergent systemic vascular associations adds another domain to this accumulating evidence, supporting the hypothesis that RPD and drusen may mark two distinct, often coexisting, macular diseases within the AMD spectrum.^10^

The load of RPD may be a useful parameter in future studies. By employing an AI algorithm, we were able to quantify the presence of RPD and drusen, enabling us to assess the impact of RPD load on CVD risk. This approach helped us mitigate the challenges posed by a relatively small sample size and low event rate. A similar approach was recently used by our group to uncover the genetic distinction between RPD and drusen.^18^

Importantly, the association between RPD load and stroke remained statistically significant after comprehensive adjustment for major cardiovascular risk factors. In our hierarchical modelling, RPD maintained significance in Model 3 (OR 1.02 per 20 lesions, 95% CI 1.00 to 1.04, p=0.042), which adjusted for age, sex, diabetes, hypertension, atrial fibrillation and lipid profile (HDL-C/LDL-C ratio). This persistence suggests RPD may capture vascular pathology beyond that reflected by conventional cardiovascular risk factors. The association remained robust in sensitivity analyses using alternative lipid parameters (total and LDL-C, p=0.047), supporting the consistency of this finding.

The statistical significance of our results diminished in models adjusting for HDL-C/LDL-C and smoking history. This may partly reflect our smaller sample size and lower event rate compared with prior studies; Ledesma-Gil *et al*, with a higher HRVD prevalence (26.5% among 200 AMD subjects), found both RPD and low HDL to be independently significant, while smoking was not. The relationship between HDL and AMD is increasingly recognised as complex. Chen *et al* recently identified a U-shaped association between HDL and AMD risk, though their study used billing codes without imaging-based phenotyping and could not distinguish between lesion types.^35^ This U-shaped relationship may be explained by differential effects on distinct lesion types. RPD are poorly visualised on colour fundus photography and require OCT or other multimodal imaging for reliable detection^28 36^; consequently, earlier large epidemiological studies using colour photography predominantly identified drusen and found high HDL to be associated with AMD risk.^37^ Thomson *et al* using OCT-based phenotyping to distinguish RPD from drusen, found the opposite association—low HDL was associated with RPD,^10^ consistent with low HDL being an established risk factor for the HRVDs implicated in RPD pathogenesis. The apparent U-shaped curve may thus reflect two distinct pathways: low HDL contributing to RPD through vascular mechanisms, and high HDL contributing to drusen formation.

A possible mechanism for the association between RPD and stroke may relate to hypoperfusion. Substantial evidence supports compromised ocular blood supply in RPD pathophysiology, including choroidal thinning,^38^ topographic alignment with choroidal watershed zones^39^ and choroidal hypoperfusion on OCT angiography.^40^ Particularly compelling is the finding by Mordechaev *et al* that internal carotid artery stenosis is associated with RPD and choroidal thinning only in the ipsilateral eye, a natural experiment in which perfusion is the single variable differing between otherwise identical eyes, strongly supporting a causal role for hypoperfusion.^41^ Fei *et al* demonstrated reduced cardiac index in patients with RPD and identified associations with valvular heart disease of at least moderate severity, suggesting that both carotid and cardiac sources of reduced perfusion may contribute.^42^

Interventional studies further support this mechanism: Berni *et al* demonstrated that carotid endarterectomy leads to sustained improvements in choroidal thickness in the ipsilateral eye,^43^ and ophthalmic artery angioplasty has shown functional benefits in late AMD.^44^ The increased mortality among individuals with RPD observed in the Beaver Dam Eye Study may reflect these global perfusion abnormalities affecting multiple organ systems.^45^

Of note, Sacconi *et al* described recently the entity pachy-RPD, characterised by a thick choroid.^46^ Further research is needed to confirm CVD associations with this entity.

We believe these findings could aid in predicting individuals’ stroke risk, complementing existing scoring systems.^47^ Although AI tools are not yet available in the clinic to quantify their numbers, clinicians can provide a rough estimate through clinical examinations or by using multimodal imaging, with a heavy RPD load alongside other CVD risk factors possibly prompting further investigation. These clinical recommendations align with those proposed previously based on the HRVD associations identified in this patient population.^31^

Our study has several limitations. First, it is a cross-sectional epidemiological study and, as such, does not establish causality. Prospective studies are necessary to validate our findings. The count of RPD lesions was not verified by human validation. The average LDL-C level was marginally high across the entire population, including the controls. This is expected in an older population like the one included in this study. Additionally, we adjusted for LDL in the logistic regression analysis. Notably, even with higher LDL levels in the control group, we observed a significant association of stroke with RPD. We did not measure choroidal thickness, which would be relevant given the proposed hypoperfusion mechanism and prior work demonstrating reduced choroidal thickness in eyes with RPD.^38 48^

In conclusion, increased RPD load was associated with elevated stroke risk, independent of traditional cardiovascular risk factors. Evaluating cardiovascular risk in patients with high RPD burden may be clinically advisable. Further prospective studies are needed to verify these findings. In particular, studies examining the development of choroidal thinning and RPD in response to the severity of carotid stenosis and cardiac insufficiency would directly test the hypoperfusion hypothesis.^31^ Such studies could also identify opportunities for earlier detection of RPD in patients with established vascular disease.

## Supplementary material

10.1136/bmjophth-2025-002481 online supplemental file 1

## Data Availability

No data are available.
