# Supplementary material for: Reticular pseudodrusen load is associated with an increased risk of stroke
Source: BMJ Open Ophthalmol. 2026 Jul 20;11(3):e002481. doi: 10.1136/bmjophth-2025-002481 (PMC13386066; doi:10.1136/bmjophth-2025-002481)
Supplement: online supplemental file 1 [file bmjophth-11-3-s001.docx]

Supplementary Table 1

|  | **Univariable** | | **Model 1**^*^ | | **Model 2**^*^ | | **Model 3**^**^ | | **Model 4**^**^ | |
| --- | --- | --- | --- | --- | --- | --- | --- | --- | --- | --- |
| **Characteristic** | **OR (95% CI)** | **p-value** | **OR (95% CI)** | **p-value** | **OR (95% CI)** | **p-value** | **OR (95% CI)** | **p-value** | **OR (95% CI)** | **p-value** |
| **Age** | 1.03 (0.97, 1.10) | 0.3 | 1.03 (0.97, 1.10) | 0.3 | 1.00 (0.94, 1.06) | >0.9 | 1.00 (0.93, 1.07) | >0.9 | 0.99 (0.93, 1.07) | 0.9 |
| **Sex** | - |  | - |  | - |  | - |  | - |  |
| Female | 1.00 |  | 1.00 |  | 1.00 |  | 1.00 |  | 1.00 (—) |  |
| Male | 1.99 (1.23, 3.28) | **0.006** | 1.98 (1.22, 3.26) | **0.006** | 1.68 (1.03, 2.81) | **0.042** | 2.02 (1.18, 3.55) | **0.012** | 1.92 (1.11, 3.40) | **0.021** |
| **Diagnosis of diabetes** | - |  | - |  | - |  | - |  | - |  |
| No | 1.00 |  | - |  | 1.00 |  | 1.00 |  | 1.00 (—) |  |
| Yes | 1.76 (0.72, 3.69) | 0.2 | - |  | 1.09 (0.43, 2.35) | 0.8 | 0.96 (0.35, 2.21) | >0.9 | 0.92 (0.34, 2.12) | 0.9 |
| **Diagnosis of hypertension** | - |  | - |  | - |  | - |  | - |  |
| No | 1.00 |  | - |  | 1.00 |  | 1.00 |  | 1.00 (—) |  |
| Yes | 3.18 (1.85, 5.77) | **<0.001** | - |  | 2.56 (1.46, 4.71) | **0.002** | 2.47 (1.36, 4.76) | **0.004** | 2.37 (1.30, 4.59) | **0.007** |
| **Diagnosis of atrial fibrillation** | - |  | - |  | - |  | - |  | - |  |
| No | 1.00 |  | - |  | 1.00 |  | 1.00 |  | 1.00 (—) |  |
| Yes | 4.86 (2.88, 8.03) | **<0.001** | - |  | 3.99 (2.32, 6.73) | **<0.001** | 3.89 (2.19, 6.77) | **<0.001** | 3.74 (2.09, 6.53) | **<0.001** |
| **HDL-C/LDL-C** | - |  | - |  | - |  | - |  | - |  |
| < 0.4 | 1.00 |  | - |  | - |  | 1.00 |  | 1.00 (—) |  |
| 0.4 to < 0.6 | 1.78 (0.99, 3.28) | 0.059 | - |  | - |  | 1.90 (1.04, 3.57) | **0.039** | 1.91 (1.04, 3.58) | **0.038** |
| >= 0.6 | 2.44 (1.20, 4.90) | **0.013** | - |  | - |  | 2.57 (1.22, 5.34) | **0.012** | 2.62 (1.24, 5.46) | **0.010** |
| **Smoking history** | - |  | - |  | - |  | - |  | - |  |
| Never | 1.00 |  | - |  | - |  | - |  | 1.00 (—) |  |
| Previous | 1.94 (1.19, 3.16) | **0.007** | - |  | - |  | - |  | 1.67 (0.97, 2.86) | 0.063 |
| Current | 0.72 (0.12, 2.39) | 0.6 | - |  | - |  | - |  | 0.65 (0.10, 2.26) | 0.6 |
| Abbreviations: CI = Confidence Interval, OR = Odds Ratio, NA | | | | | | | | | | |
| OR: odds ratio, CI: confidence interval, RPD: reticular pseudodrusen, HDL-C: high-density lipoprotein cholesterol, LDL-C: low-density lipoprotein cholesterol. | | | | | | | | | | |
| OR > 1.00 imply greater odds of stroke. Bold p-values represent statistically significant results. | | | | | | | | | | |
| ^*^Number of observations = 2,010 | | | | | | | | | | |
| ^**^Number of observations = 1,812 | | | | | | | | | | |

**Supplementary Table 1. Hierarchical logistic regression models for stroke risk without reticular pseudodrusen load.**

Raw and mutually adjusted odds ratios (OR) for stroke, excluding RPD load as a predictor. Model 1 adjusts for age, sex, and number of drusen. Model 2 adjusts as Model 1 plus diagnosis of diabetes, hypertension, and atrial fibrillation. Model 3 adjusts as Model 2 plus HDL-C/LDL-C ratio, and Model 4 adjusts as Model 3 plus smoking history. These models are presented for comparison with the main analysis (Table 2) to demonstrate the incremental predictive value of RPD load. OR = odds ratio; CI = confidence interval; HDL-C = high-density lipoprotein cholesterol; LDL-C = low-density lipoprotein cholesterol.

Supplementary Table 2

|  | **Univariable** | | **Model 1**^*^ | | **Model 2**^*^ | | **Model 3**^**^ | | **Model 4**^**^ | |
| --- | --- | --- | --- | --- | --- | --- | --- | --- | --- | --- |
| **Characteristic** | **OR (95% CI)** | **p-value** | **OR (95% CI)** | **p-value** | **OR (95% CI)** | **p-value** | **OR (95% CI)** | **p-value** | **OR (95% CI)** | **p-value** |
| **Age** | 1.07 (1.02, 1.13) | **0.010** | 1.07 (1.01, 1.13) | **0.017** | 1.04 (0.99, 1.10) | 0.13 | 1.05 (0.99, 1.12) | 0.091 | 1.05 (0.99, 1.12) | 0.12 |
| **Sex** | - |  | - |  | - |  | - |  | - |  |
| Female | 1.00 |  | 1.00 |  | 1.00 |  | 1.00 |  | 1.00 (—) |  |
| Male | 3.05 (2.04, 4.66) | **<0.001** | 3.01 (2.01, 4.60) | **<0.001** | 2.52 (1.67, 3.88) | **<0.001** | 2.82 (1.80, 4.52) | **<0.001** | 2.52 (1.60, 4.07) | **<0.001** |
| **Diagnosis of diabetes** | - |  | - |  | - |  | - |  | - |  |
| No | 1.00 |  | - |  | 1.00 |  | 1.00 |  | 1.00 (—) |  |
| Yes | 4.01 (2.35, 6.60) | **<0.001** | - |  | 2.45 (1.40, 4.13) | **0.001** | 2.05 (1.10, 3.64) | **0.018** | 1.92 (1.03, 3.42) | **0.033** |
| **Diagnosis of hypertension** | - |  | - |  | - |  | - |  | - |  |
| No | 1.00 |  | - |  | 1.00 |  | 1.00 |  | 1.00 (—) |  |
| Yes | 4.07 (2.58, 6.69) | **<0.001** | - |  | 3.08 (1.93, 5.12) | **<0.001** | 2.84 (1.73, 4.87) | **<0.001** | 2.69 (1.64, 4.62) | **<0.001** |
| **Diagnosis of atrial fibrillation** | - |  | - |  | - |  | - |  | - |  |
| No | 1.00 |  | - |  | 1.00 |  | 1.00 |  | 1.00 (—) |  |
| Yes | 2.98 (1.88, 4.60) | **<0.001** | - |  | 2.09 (1.29, 3.32) | **0.002** | 2.07 (1.23, 3.37) | **0.005** | 1.97 (1.17, 3.23) | **0.009** |
| **HDL-C/LDL-C** | - |  | - |  | - |  | - |  | - |  |
| < 0.4 | 1.00 |  | - |  | - |  | 1.00 |  | 1.00 (—) |  |
| 0.4 to < 0.6 | 1.72 (1.09, 2.76) | **0.021** | - |  | - |  | 1.72 (1.07, 2.80) | **0.028** | 1.74 (1.08, 2.83) | **0.025** |
| >= 0.6 | 1.99 (1.11, 3.51) | **0.018** | - |  | - |  | 2.02 (1.09, 3.66) | **0.022** | 2.02 (1.09, 3.68) | **0.023** |
| **Smoking history** | - |  | - |  | - |  | - |  | - |  |
| Never | 1.00 |  | - |  | - |  | - |  | 1.00 (—) |  |
| Previous | 2.76 (1.84, 4.14) | **<0.001** | - |  | - |  | - |  | 1.95 (1.25, 3.05) | **0.003** |
| Current | 3.72 (1.82, 7.06) | **<0.001** | - |  | - |  | - |  | 2.52 (1.12, 5.22) | **0.018** |
| Abbreviations: CI = Confidence Interval, OR = Odds Ratio, NA | | | | | | | | | | |
| OR: odds ratio, CI: confidence interval, RPD: reticular pseudodrusen, HDL-C: high-density lipoprotein cholesterol, LDL-C: low-density lipoprotein cholesterol. | | | | | | | | | | |
| OR > 1.00 imply greater odds of stroke. Bold p-values represent statistically significant results. | | | | | | | | | | |
| ^*^Number of observations = 2,010 | | | | | | | | | | |
| ^**^Number of observations = 1,812 | | | | | | | | | | |

**Supplementary Table 2. Hierarchical logistic regression models for myocardial infarction risk without reticular pseudodrusen load.**

Raw and mutually adjusted odds ratios (OR) for myocardial infarction, excluding RPD load as a predictor. Model 1 adjusts for age, sex, and number of drusen. Model 2 adjusts as Model 1 plus diagnosis of diabetes, hypertension, and atrial fibrillation. Model 3 adjusts as Model 2 plus HDL-C/LDL-C ratio, and Model 4 adjusts as Model 3 plus smoking history. These models are presented for comparison with Supplementary Table 3 to demonstrate that RPD load does not provide incremental predictive value for myocardial infarction, in contrast to its association with stroke. OR = odds ratio; CI = confidence interval; HDL-C = high-density lipoprotein cholesterol; LDL-C = low-density lipoprotein cholesterol.

Supplementary Table 3

| **Characteristic** | **OR (95% CI)** | **p-value** |
| --- | --- | --- |
| **RPD (per 20)** | 1.02 (1.00, 1.04) | **0.047** |
| **Age** | 1.00 (0.94, 1.07) | >0.9 |
| **Sex** | - |  |
| Female | 1.00 |  |
| Male | 1.48 (0.83, 2.68) | 0.2 |
| **Diagnosis of diabetes** | - |  |
| No | 1.00 |  |
| Yes | 0.70 (0.25, 1.63) | 0.4 |
| **Diagnosis of hypertension** | - |  |
| No | 1.00 |  |
| Yes | 2.13 (1.16, 4.13) | **0.018** |
| **Diagnosis of atrial fibrillation** | - |  |
| No | 1.00 |  |
| Yes | 3.80 (2.13, 6.63) | **<0.001** |
| **Total cholesterol** | 1.46 (0.67, 3.05) | 0.3 |
| **LDL cholesterol** | 0.31 (0.12, 0.86) | **0.021** |
| Abbreviations: CI = Confidence Interval, OR = Odds Ratio | | |
| OR: odds ratio, CI: confidence interval, RPD: reticular pseudodrusen, LDL: low-density lipoprotein. | | |
| OR > 1.00 imply greater odds of stroke. Bold p-values represent statistically significant results. | | |

**Supplementary Table 3. Sensitivity analysis: Stroke risk model using total and LDL cholesterol.**

Fully adjusted logistic regression model for stroke risk using alternative lipid parameterization. This model adjusts for age, sex, drusen count, diabetes, hypertension, atrial fibrillation, total cholesterol (continuous), and LDL cholesterol (continuous), instead of the HDL-C/LDL-C ratio used in the main analysis. This sensitivity analysis demonstrates the robustness of the RPD-stroke association to different specifications of lipid risk factors. RPD = reticular pseudodrusen; OR = odds ratio; CI = confidence interval; LDL-C = low-density lipoprotein cholesterol.
